# Turkish Adaptation and Psychometric Evaluation of the Measure of Processes of Care for Service Providers in Pediatric Care and Rehabilitation

**DOI:** 10.3390/children13101389

**Published:** 2026-10-13

**Authors:** Cemil Özal, Duygu Türker, Mintaze Kerem Gunel

**Affiliations:** 1 Faculty of Physical Therapy and Rehabilitation, Hacettepe University, Ankara 06100, Türkiye; cemil.ozal@hacettepe.edu.tr (C.Ö.); mintaze@hacettepe.edu.tr (M.K.G.); 2 Gülhane Faculty of Physiotherapy and Rehabilitation, University of Health Sciences, Ankara 06018, Türkiye

**Keywords:** family-centered services, MPOC-SP, psychometric validation, reliability, validity, pediatric rehabilitation, cross-cultural adaptation

## Abstract

**Highlights:**

**What are the main findings?**

- The findings supported the hypothesized four-factor structure of the Turkish MPOC-SP, corresponding to Showing Interpersonal Sensitivity, Treating People Respectfully, Communicating Specific Information, and Providing General Information.
- The four subscales demonstrated high internal consistency and good to excellent test–retest reliability, although small systematic test–retest differences were observed for SIS and CSI.

**What are the implications of the main findings?**

- The findings provide initial psychometric evidence supporting interpretation of the Turkish MPOC-SP at the level of its four established subscales; item-level ceiling effects should be considered when interpreting very high scores.
- The Turkish MPOC-SP may be useful for assessing service providers’ perceptions of family-centered services across its four established dimensions; however, further research is needed to establish external construct validity, measurement invariance, responsiveness, and clinically meaningful change, particularly for longitudinal applications.

**Abstract:**

**Background/Objectives**: Family-centered services (FCSs) are a key approach in pediatric healthcare and rehabilitation that promotes collaboration between service providers and families. The Measure of Processes of Care for Service Providers (MPOC-SP) is widely used to evaluate family-centered services from the provider perspective; however, no validated Turkish version is available. This study aimed to translate and culturally adapt the MPOC-SP into Turkish and to examine its factor structure, internal consistency, test–retest reliability, measurement error, and supplementary model-based construct indices among Turkish-speaking service providers working in pediatric healthcare, disability, and rehabilitation settings. **Methods**: The MPOC-SP was translated and culturally adapted following a structured cross-cultural adaptation process. A total of 267 service providers from multiple professional disciplines, institutional settings, and 15 provinces of Türkiye participated in the psychometric evaluation. Internal consistency was assessed using Cronbach’s alpha and McDonald’s omega. Test–retest reliability was evaluated in 86 participants using ICC(2,1), and measurement error was examined using the standard error of measurement (SEM) and minimal detectable change at the 95% confidence level (MDC95). The hypothesized four-factor structure was evaluated in 267 participants using confirmatory factor analysis based on polychoric correlations and diagonally weighted least squares (DWLS). Composite reliability, average variance extracted, the Fornell–Larcker criterion, and the heterotrait–monotrait ratio were examined as supplementary model-based indices. Institutional-group comparisons were exploratory. **Results**: The four subscales demonstrated high internal consistency, with Cronbach’s alpha coefficients ranging from 0.884 to 0.968 and McDonald’s omega coefficients from 0.885 to 0.968. Test–retest reliability was good to excellent (ICC[2,1] = 0.819–0.963), although small systematic decreases were observed for SIS and CSI. SEM values ranged from 0.228 to 0.328 and MDC95 values from 0.633 to 0.908. The four-factor model showed a good fit (χ^2^(318) = 463.24, CFI = 0.998, TLI = 0.998, RMSEA = 0.041, 90% CI [0.033–0.049], SRMR = 0.057), with standardized factor loadings ranging from 0.761 to 0.933. Composite reliability ranged from 0.899 to 0.976, AVE from 0.640 to 0.800, and HTMT values from 0.372 to 0.664. No substantial ceiling effects were observed at the subscale level; however, 14 of 27 items exceeded the predefined 15% criterion for item-level ceiling effects. **Conclusions**: The findings provide initial evidence supporting the four-factor structure and reliability of the Turkish MPOC-SP in this sample of service providers. Interpretation should focus on the four established subscales rather than an overall total score. Further studies in independent and more balanced samples are needed to examine external construct validity, measurement invariance, responsiveness, and clinically meaningful change.

## 1. Introduction

Family-centered services (FCSs) are widely recognized as a core framework for the organization and delivery of healthcare and rehabilitation services for children with disabilities or chronic conditions and their families [1,2]. Within the FCS approach, families are viewed as active partners in care, with service delivery grounded in collaboration, respect, information sharing, and shared decision-making [3]. FCS is also consistent with the International Classification of Functioning, Disability, and Health (ICF), which conceptualizes functioning and disability through interactions among health conditions, activities, participation, and contextual factors, including the family environment. Accordingly, FCS is particularly relevant to pediatric rehabilitation, where responsiveness to family priorities and support for meaningful participation are central components of service delivery [4,5,6,7].

As FCS has become an established approach to pediatric service delivery, the need for valid and reliable instruments to evaluate how these principles are implemented in practice has increased. Within this context, the Measure of Processes of Care (MPOC) was developed at the CanChild Centre for Childhood Disability Research to assess family-centered service processes. The MPOC family of instruments focuses on care processes rather than clinical outcomes and provides a structured means of evaluating the extent to which services reflect FCS [8].

The original MPOC instruments primarily capture parents’ perceptions of family-centered service delivery. To provide a complementary service-provider perspective, the Measure of Processes of Care for Service Providers (MPOC-SP) was developed as a 27-item self-report instrument assessing providers’ reported family-centered practices. The MPOC-SP comprises four established dimensions: Showing Interpersonal Sensitivity (SIS), Treating People Respectfully (TPR), Communicating Specific Information (CSI), and Providing General Information (PGI). These dimensions represent related but distinct aspects of family-centered service delivery and form the conceptual basis for evaluating the four-factor structure of the instrument [9,10].

The MPOC-SP has been used in pediatric rehabilitation and related healthcare settings to evaluate FCS delivery from the provider perspective. Previous studies have applied the instrument across different service contexts and professional groups, supporting its relevance for examining providers’ perceptions of FCSs [11,12].

The MPOC-SP has been translated and culturally adapted for use in several languages and healthcare systems. However, previous validation studies have differed in their psychometric approaches. Studies of the Dutch, Chinese, and Korean versions primarily examined internal consistency, test–retest reliability, item–subscale relationships, and other aspects of construct validity, whereas factor-analytic evaluation has been undertaken in some other adaptations. Overall, these studies have provided support for the use of the MPOC-SP across different cultural contexts, although the strength and type of evidence for its measurement structure have not been uniform. These differences highlight the importance of directly examining the hypothesized four-factor structure when adapting the MPOC-SP to a new language and cultural context [12,13,14,15,16,17,18].

In Türkiye, increasing attention has been directed toward FCS delivery in pediatric healthcare and rehabilitation. Although Turkish versions of parent-reported MPOC instruments are available, to our knowledge, no validated Turkish-language instrument measures a sufficiently comparable service provider–reported construct of family-centered service delivery. A culturally adapted Turkish version of the MPOC-SP would therefore complement the existing family-reported measures by enabling FCS processes to be examined from the service-provider perspective. Establishing its measurement properties in Turkish-speaking service providers is necessary before the instrument can be appropriately used in the Turkish context [19,20,21,22].

Therefore, this study aimed to translate and culturally adapt the MPOC-SP into Turkish and to examine its measurement properties among Turkish-speaking service providers working in pediatric healthcare, disability, and rehabilitation settings. Specifically, we evaluated the hypothesized four-factor structure of the Turkish MPOC-SP, internal consistency of the four subscales, test–retest reliability, and measurement error. Based on the established conceptual structure of the original MPOC-SP, the Turkish version was expected to reproduce four correlated but distinct dimensions—SIS, TPR, CSI, and PGI—with items loading primarily on their intended factors. Associations among the four latent dimensions and supplementary model-based indices of construct reliability, variance extraction, and factor distinctiveness were also examined. In addition, MPOC-SP subscale scores were compared across institutional settings as an exploratory analysis. Because no independent criterion or a priori evidence was available to establish expected differences in FCS across these settings, no directional institutional-group hypotheses were specified, and these comparisons were not considered tests of known-groups validity.

## 2. Materials and Methods

### 2.1. Study Design

This study was designed as a methodological study aimed at translating, culturally adapting, and examining the measurement properties of the Turkish version of the MPOC-SP. The study followed internationally accepted guidelines for cross-cultural adaptation and psychometric validation of self-report instruments [23]. The terminology and reporting of measurement properties in the present study were aligned with the COSMIN framework, including structural validity, internal consistency, reliability, measurement error, and hypothesis testing for construct validity [24].

### 2.2. Instrument

The MPOC-SP is a self-report questionnaire developed to assess service providers’ perceptions of the extent to which their practices reflect FCS principles. The instrument focuses on care processes rather than clinical outcomes and consists of 27 items rated from 1 to 7, with higher scores indicating a greater degree of FCS delivery; 0 indicates ‘not applicable’ [9,10]. Consistent with MPOC-SP scoring, responses coded as 0 were excluded from the calculation of the corresponding subscale mean. Each subscale score was calculated as the mean of the valid responses to the items belonging to that subscale; no overall MPOC-SP total score was calculated. At baseline, all 267 participants provided valid responses to all 27 items. At retest, one response coded as 0 occurred for one TPR item in one participant; that participant’s TPR subscale score was calculated from the remaining valid TPR items.

The MPOC-SP is divided into four dimensions: SIS, comprising 10 items; TPR, with 9 items; CSI, with 3 items; and PGI, with 5 items. Items are scored from 1 to 7, while a response of 0 indicates “not applicable”. Subscale scores are calculated by averaging the applicable item scores within each dimension, with higher scores indicating a greater frequency of family-centered behaviors as perceived by service providers. In the present study, psychometric interpretation was restricted to these four established subscale scores [9,10].

### 2.3. Translation and Cultural Adaptation Procedure

To adapt the MPOC-SP into Turkish, written permission was obtained from the CanChild Centre, which holds the publication rights to the original scale.

The translation and cultural adaptation of the MPOC-SP into Turkish were conducted using a standardized forward–backward translation methodology in accordance with internationally accepted guidelines for cross-cultural adaptation of self-report instruments [23]. Initially, the original English version was independently translated into Turkish by the first and second authors, who are bilingual researchers whose native language is Turkish and who each have at least 15 years of experience in pediatric rehabilitation. The two forward translations were compared and reconciled through consensus to produce a preliminary Turkish version. This version was then independently back-translated into English by two professional translators who were native English speakers, proficient in Turkish, independent of the research team, and blinded to both the subject matter and the original English questionnaire. The back-translations were compared with the original instrument to assess conceptual equivalence, and the original developers were consulted during this process.

An expert committee consisting of nine members—a physician, a physiotherapist, an occupational therapist, a speech and language therapist, a special education teacher, a nurse, a psychologist, and two Turkish linguists—reviewed all translated versions to evaluate semantic, idiomatic, experiential, and conceptual equivalence. The healthcare and rehabilitation professionals had at least 15 years of experience in pediatric healthcare and rehabilitation, while the linguists had expertise in language and translation. Any discrepancies identified during the review process were resolved through consensus. Minor modifications were made to three items to improve linguistic clarity and cultural appropriateness without altering the conceptual meaning of the original instrument. These modifications involved the wording corresponding to “anticipate” in Item 9, “community’s resource library” in Item 25, and “dating and sexuality” in Item 27.

The pre-final Turkish version of the MPOC-SP was pilot-tested with 10 service providers, including two physicians, two physiotherapists, two occupational therapists, one speech and language therapist, one special education teacher, one nurse, and one psychologist, all of whom were working in pediatric healthcare or rehabilitation settings. Item-by-item verbal probing was used to explore comprehension and identify unclear expressions (e.g., “What do you understand from this item?” and “Is there any expression that is unclear?”). Feedback from this cognitive debriefing stage was reviewed by the research team, and the final Turkish version was prepared after confirming comprehensibility and conceptual consistency. Feedback was collected through written comments. Based on the participants’ feedback, no further revisions were required, and the final Turkish version was approved for psychometric evaluation. The expert committee review was qualitative; no item-level or scale-level content validity indices were calculated.

### 2.4. Participants

Participants were recruited through professional associations, hospitals, rehabilitation centers, and professional WhatsApp networks. Invitations were distributed individually rather than through an unrestricted open link. Approximately 450 potentially eligible service providers were invited to participate. The Google Forms setting ‘Limit to 1 response’ was enabled to reduce duplicate submissions. Eligibility was determined at the beginning of the questionnaire. Eligible participants were service providers from the specified professional disciplines who were currently working in pediatric healthcare, disability, rehabilitation, or related service settings in Türkiye. They were required to be able to complete the Turkish-language questionnaire and to be actively providing services to children and their families. All participants worked in pediatric practice; therefore, pediatric service provision characterized the entire study sample rather than being defined by a minimum weekly caseload threshold. No minimum number of years of professional experience was specified as an eligibility criterion. Professional discipline, institutional setting, and years of professional experience were recorded as participant characteristics.

The target sample size was considered in relation to the complexity of the 27-item, four-factor measurement model rather than relying solely on a fixed participant-to-item ratio. Simulation-based research has shown that sample-size requirements for CFA/SEM vary substantially according to model complexity, factor loadings, the number of indicators and factors, missing data, and other data characteristics, and therefore cannot be adequately determined by a single participant-to-item rule [25]. The final baseline sample of 267 participants provided complete responses to all 27 items and was used for the CFA. Given the model complexity, the complete baseline data, and the use of an estimator appropriate for ordinal responses, this sample was considered reasonable for the planned factor-analytic evaluation, while recognizing that CFA sample-size requirements are model- and data-dependent.

Eligible participants included physiotherapists, occupational therapists, speech and language therapists, psychologists, special education/child development professionals, physicians, and nurses who were directly involved in pediatric services. Participants who did not meet the eligibility criteria or who submitted incomplete questionnaires or participant-information data were excluded from the psychometric sample.

Participation was voluntary, and informed consent was obtained from all participants prior to data collection in accordance with the Declaration of Helsinki. Ethical approval for the study was obtained from the Unit Research Ethics Board of Hacettepe University Physical Therapy and Rehabilitation Faculty (Approval No: FTREK25/109, Date: 24 July 2025).

### 2.5. Data Collection

Data were collected between September 2025 and November 2025 using an online questionnaire administered through Google Forms (Google LLC, Mountain View, CA, USA; https://forms.google.com, accessed on 22 September 2026). Participants completed the Turkish version of the MPOC-SP electronically after providing informed consent.

For test–retest reliability, email addresses were collected at baseline solely to contact participants selected for the test–retest assessment and to match repeated measurements. To maintain confidentiality, participants were assigned study codes, and identifying information was removed from the analytical dataset before statistical analysis. Email addresses were not included in the dataset used for psychometric analyses. Based on the chronological order of baseline completion, every third respondent was invited to complete the Turkish MPOC-SP again two weeks later, resulting in a total of 90 invited participants. Of those invited, 86 completed the retest assessment. No relevant changes in professional role or service conditions were reported during the two-week interval. The test–retest subsample was compared with baseline-only participants on demographic, professional, institutional, and baseline MPOC-SP characteristics to examine potential selection differences.

### 2.6. Statistical Analysis

Statistical analyses were performed using IBM SPSS Statistics for Windows, Version 25.0 (IBM Corp., Armonk, NY, USA) and R version 4.3.3 (R Foundation for Statistical Computing, Vienna, Austria). Descriptive statistics, distributional analyses, Cronbach’s alpha, test–retest reliability, measurement error analyses, and institutional-group comparisons were performed using IBM SPSS Statistics 25.0. McDonald’s omega and polychoric correlation analyses were performed in R using the psych package (version 2.4.3). Confirmatory factor analysis (CFA) of the ordinal item data was performed in R using the lavaan package (version 0.6-18), based on polychoric correlations and diagonally weighted least squares (DWLS). Composite reliability (CR), average variance extracted (AVE), the Fornell–Larcker criterion, and heterotrait–monotrait ratios (HTMT) were calculated from the standardized CFA solution. Parametric model-based bootstrap confidence intervals for latent factor correlations were obtained using 20,000 replications. Statistical significance was set at *p* < 0.05 unless otherwise specified.

Descriptive and reliability analyses were conducted for the four established MPOC-SP subscales.

Prior to analysis, the dataset was screened for completeness and consistency. Responses coded as 0 (“not applicable”) were excluded from the calculation of the relevant subscale mean rather than treated as substantive scores. At baseline, all 267 participants had valid responses for all 27 MPOC-SP items; therefore, the effective CFA sample was N = 267. In the retest dataset, one 0 response occurred within the TPR subscale; that participant’s TPR score was calculated from the remaining applicable items.

The distribution of continuous variables was assessed using the Shapiro–Wilk test together with visual inspection of histograms and Q–Q plots. Descriptive statistics were presented as mean ± standard deviation (SD) for normally distributed variables and as median (interquartile range) for non-normally distributed variables. Categorical variables were summarized using frequencies and percentages.

Internal consistency of each MPOC-SP subscale was evaluated using Cronbach’s alpha and McDonald’s omega coefficients. Corrected item–subscale correlations were calculated to examine the association of each item with its intended subscale. Floor and ceiling effects were calculated as the percentage of participants obtaining the lowest and highest possible scores, respectively, and were considered substantial when more than 15% of participants achieved the minimum or maximum possible score.

Test–retest reliability was evaluated using the intraclass correlation coefficient based on a two-way random-effects model for absolute agreement with single measurements (ICC[2,1]), and 95% confidence intervals were reported. ICC values < 0.50 were interpreted as poor, 0.50–0.75 as moderate, 0.75–0.90 as good, and >0.90 as excellent reliability [26]. Systematic differences between test and retest scores were also examined, with mean differences calculated as test minus retest (Test − Retest); 95% confidence intervals and corresponding *p*-values were reported. Measurement error was quantified using the standard error of measurement (SEM), calculated as SDpooled × √(1 − ICC), where SDpooled represents the pooled standard deviation of test and retest scores. The minimal detectable change at the 95% confidence level was calculated as MDC95 = SEM × 1.96 × √2 [27].

The hypothesized four-factor structure of the Turkish MPOC-SP was examined using CFA. Because the MPOC-SP items are measured on an ordinal response scale and multivariate normality was not supported, the CFA was based on a polychoric correlation matrix and estimated using diagonally weighted least squares (DWLS), an approach appropriate for ordinal indicators [28]. The model specified four correlated latent factors corresponding to SIS, TPR, CSI, and PGI, with each item loading only on its intended factor. No cross-loadings, correlated residuals, or post hoc model modifications were introduced. Model fit was evaluated using the chi-square statistic (χ^2^), chi-square-to-degrees-of-freedom ratio (χ^2^/df), comparative fit index (CFI), Tucker–Lewis index (TLI), root mean square error of approximation (RMSEA) with its 90% confidence interval, and standardized root mean square residual (SRMR). Standard errors and 95% confidence intervals were obtained for the standardized factor loadings.

Composite reliability (CR) and average variance extracted (AVE) were calculated from the standardized CFA solution and were treated as supplementary model-based indices of construct reliability and variance extraction. Factor distinctiveness was additionally examined using the Fornell–Larcker criterion and the heterotrait–monotrait ratio of correlations (HTMT). Approximate 95% confidence intervals for latent factor correlations were obtained using a parametric model-based bootstrap with 20,000 replications, conditional on the fitted four-factor latent correlation structure and the observed CFA sample size (N = 267); percentile-based 95% confidence intervals were derived from the bootstrap distributions. These indices were interpreted as supplementary evidence regarding the internal measurement model and were not considered substitutes for external convergent or discriminant validity.

MPOC-SP subscale scores were compared across institutional settings as an exploratory analysis. Because no independent criterion or a priori evidence was available to establish expected differences in family-centered service delivery across institutional settings, these comparisons were not considered tests of known-groups validity. As distributional assumptions were not satisfied across all institutional groups, Kruskal–Wallis tests were used for overall comparisons. To control multiplicity across the four predefined MPOC-SP subscales, a Bonferroni-adjusted significance level of α = 0.0125 (0.05/4) was applied to the omnibus Kruskal–Wallis tests. Epsilon-squared was calculated as ε^2^ = (H − k + 1)/(N − k), where H is the Kruskal–Wallis statistic, k is the number of groups, and N is the total sample size; negative estimates were reported as zero. When an omnibus test remained significant after this correction, pairwise institutional comparisons were performed using Mann–Whitney U tests with Bonferroni adjustment across the six possible pairwise comparisons. Pairwise effect sizes were calculated as r = |Z|/√N, where N is the number of participants in the two groups being compared. Given the association between professional discipline and institutional setting, supplementary adjusted analyses controlling for profession, age, and years of professional experience were also performed.

## 3. Results

### 3.1. Participant Characteristics

Approximately 450 potentially eligible service providers were invited to participate, and 283 submitted the questionnaire (response proportion among those approached, 62.9%). Because recruitment was based on nonprobability sampling rather than a population-based sampling frame, this proportion should not be interpreted as a population-level response rate. Sixteen responses were excluded: nine because of incomplete MPOC-SP data, three because of incomplete participant information, two because participants did not meet the eligibility criteria, and two for overlapping reasons, leaving 267 eligible participants for the baseline psychometric analyses (94.3% of respondents; 59.3% of those approached). No baseline item-level missing or not-applicable responses were present among these 267 participants.

Participants had a mean age of 33.13 ± 8.00 years and a mean of 10.17 ± 7.84 years of professional experience; 74.5% were female. The sample included physiotherapists, occupational therapists, speech and language therapists, psychologists, special education/child development professionals, physicians, and nurses. Participants worked in special education and rehabilitation centers (n = 146, 54.7%), public hospitals (n = 60, 22.5%), private hospitals (n = 34, 12.7%), and private practice (n = 27, 10.1%).

Participants were recruited from 15 provinces: Istanbul (n = 61, 22.8%), Ankara (n = 59, 22.1%), Izmir (n = 29, 10.9%), Bursa (n = 16, 6.0%), Antalya (n = 14, 5.2%), Konya (n = 12, 4.5%), Adana (n = 12, 4.5%), Kocaeli (n = 10, 3.7%), Şanlıurfa (n = 9, 3.4%), Gaziantep (n = 9, 3.4%), Diyarbakır (n = 8, 3.0%), Mersin (n = 8, 3.0%), Hatay (n = 7, 2.6%), Trabzon (n = 7, 2.6%), and Giresun (n = 6, 2.2%). Detailed participant characteristics are presented in Table 1.

### 3.2. Internal Consistency and Item Analysis

The four Turkish MPOC-SP subscales demonstrated high internal consistency. Cronbach’s alpha coefficients were 0.968 for SIS, 0.941 for TPR, 0.896 for CSI, and 0.884 for PGI, while McDonald’s omega coefficients were 0.968, 0.941, 0.896, and 0.885, respectively. Corrected item–subscale correlations ranged from 0.807 to 0.895 for SIS, 0.710 to 0.832 for TPR, 0.786 to 0.813 for CSI, and 0.684 to 0.779 for PGI.

No substantial floor or ceiling effects were observed at the subscale level. Ceiling effects were 2.25% for SIS, 0.37% for TPR, 5.62% for CSI, and 1.12% for PGI, while floor effects ranged from 0% to 0.37%. At the item level, however, 14 of the 27 items exceeded the predefined 15% criterion for a ceiling effect, with the highest item-level ceiling effect being 25.09%.

Item-level descriptive statistics, floor and ceiling effects, and corrected item–subscale correlations are presented in Table 2.

### 3.3. Test–Retest Reliability

The test–retest subsample (n = 86) was compared with participants who completed the baseline assessment only (n = 181). No statistically significant differences were observed in age, professional experience, sex, profession, institutional setting, SIS, or PGI. However, small baseline differences were observed for TPR (*p* = 0.019, r = 0.144) and CSI (*p* = 0.003, r = 0.181). These findings indicate that the test–retest subsample was generally comparable to the baseline-only participants, although complete comparability cannot be assumed for all MPOC-SP subscales.

ICC(2,1) values ranged from 0.819 to 0.963 across the four subscales, indicating good to excellent relative reliability. The highest ICC was observed for SIS (ICC = 0.963, 95% CI: 0.932–0.979), whereas the lowest was observed for TPR (ICC = 0.819, 95% CI: 0.718–0.885). Significant test–retest differences were observed for SIS (mean difference = 0.129, 95% CI: 0.065–0.193, *p* < 0.001) and CSI (mean difference = 0.213, 95% CI: 0.124–0.303, *p* < 0.001), with lower mean scores at retest. No significant systematic differences were observed for TPR (mean difference = 0.026, 95% CI: −0.073–0.125, *p* = 0.601) or PGI (mean difference = 0.042, 95% CI: −0.027–0.111, *p* = 0.231).

SEM values ranged from 0.228 to 0.328, and MDC95 values ranged from 0.633 to 0.908 across the four subscales. Detailed test–retest reliability and measurement-error estimates are presented in Table 3.

### 3.4. Factor Structure and Model-Based Indices

The CFA was conducted using data from all 267 participants, for whom complete responses were available for all 27 MPOC-SP items at baseline. Item-level distributional characteristics were also examined. Across the 27 MPOC-SP items, skewness ranged from −1.181 to −0.382, and excess kurtosis ranged from −0.253 to 1.770. All items showed negative skewness, consistent with the concentration of responses toward the higher response categories observed in the item-level distributions. Multivariate normality was further assessed using Mardia’s tests and was not supported (Mardia’s multivariate skewness = 137.996, χ^2^(3654) = 6140.84, *p* < 0.001; multivariate kurtosis = 913.111, z = 26.86, *p* < 0.001). Accordingly, the hypothesized correlated four-factor model was evaluated using a polychoric correlation-based DWLS approach. The four-factor model showed a good fit to the data: χ^2^(318) = 463.24, χ^2^/df = 1.46, CFI = 0.998, TLI = 0.998, RMSEA = 0.041 (90% CI: 0.033–0.049), and SRMR = 0.057. The CFA model-fit indices are presented in Table 4. Standardized factor loadings ranged from 0.761 to 0.933, with all items loading strongly on their intended factors. The 95% confidence intervals for the standardized factor loadings did not include zero (Table 5). The estimated latent factor correlations were 0.385 for SIS–TPR (approximate 95% bootstrap CI: 0.277–0.483), 0.379 for SIS–CSI (0.271–0.478), 0.420 for SIS–PGI (0.316–0.517), 0.679 for TPR–CSI (0.611–0.739), 0.525 for TPR–PGI (0.434–0.608), and 0.642 for CSI–PGI (0.567–0.709).

All standardized factor loadings exceeded 0.70, indicating strong item–factor relationships within the specified four-factor model. Detailed standardized factor loadings for the 27 items are presented in Table 5. No cross-loadings or correlated residuals were specified in the model.

Composite reliability values ranged from 0.899 to 0.976, and AVE values ranged from 0.640 to 0.800. For each factor, the square root of the AVE exceeded its correlations with the remaining factors, satisfying the Fornell–Larcker criterion. HTMT values ranged from 0.372 to 0.664, with all values below 0.85 (Table 6A).

Factor correlations ranged from 0.379 to 0.679, with the strongest correlation observed between TPR and CSI (r = 0.679). Factor correlations and square roots of AVE are presented in Table 6B.

### 3.5. Exploratory Institutional-Group Comparisons

Exploratory comparisons of MPOC-SP subscale scores across institutional settings showed no significant differences for SIS (H = 5.875, *p* = 0.118, ε^2^ = 0.011), TPR (H = 1.876, *p* = 0.599, ε^2^ = 0.000), or CSI (H = 1.847, *p* = 0.605, ε^2^ = 0.000). Using the Bonferroni-adjusted omnibus significance level of α = 0.0125, a significant institutional difference was observed for PGI (H = 16.841, *p* < 0.001, ε^2^ = 0.053) (Table 7). The distribution of professional disciplines across institutional settings is presented in Table 8.

Bonferroni-adjusted pairwise comparisons for PGI showed higher scores among participants working in private practice than among those working in special education and rehabilitation centers (U = 1296.0, adjusted *p* = 0.028, r = 0.215) and public hospitals (U = 395.5, adjusted *p* < 0.001, r = 0.408). The remaining comparisons were not statistically significant after Bonferroni adjustment: special education and rehabilitation centers versus public hospitals (U = 5333.5, adjusted *p* = 0.083, r = 0.171), special education and rehabilitation centers versus private hospitals (U = 2495.0, adjusted *p* = 1.000, r = 0.003), public hospitals versus private hospitals (U = 808.5, adjusted *p* = 0.576, r = 0.172), and private hospitals versus private practice (U = 302.5, adjusted *p* = 0.138, r = 0.291). Professional discipline was associated with institutional setting (χ^2^(18) = 223.05, *p* < 0.001, Cramér’s V = 0.528). In analyses adjusted for profession, age, and years of professional experience, the institutional effect on PGI remained statistically significant (*p* = 0.011 in the conventional model and *p* = 0.013 using HC3 robust standard errors).

## 4. Discussion

The present study translated, culturally adapted, and evaluated the measurement properties of the Turkish version of the MPOC-SP in a heterogeneous sample of professionals working in pediatric healthcare, rehabilitation, and educational services. Overall, the findings supported the hypothesized four-factor structure of the Turkish MPOC-SP and indicated high internal consistency and good to excellent test–retest reliability across its four subscales. Model-based indices provided additional evidence regarding the internal measurement structure. These findings add to the broader MPOC literature demonstrating the value of MPOC-based instruments for evaluating family-centered service processes from the perspectives of families and service providers [9].

The four MPOC-SP subscales demonstrated high internal consistency, with closely comparable Cronbach’s alpha and McDonald’s omega coefficients. These findings are broadly consistent with previous cross-cultural evaluations of the MPOC-SP. The Spanish version demonstrated satisfactory internal consistency across its dimensions [13], while the Korean version also showed acceptable to high internal consistency across the four subscales [15]. The particularly high internal consistency observed for SIS should nevertheless be interpreted with some caution, as very high coefficients may also reflect overlap or redundancy among items [29]. Moreover, high internal consistency alone does not establish unidimensionality [29,30]. Accordingly, the similar alpha and omega coefficients should be interpreted as complementary evidence of internal consistency and not as independent evidence of the factor structure, which is primarily supported by the CFA findings.

Test–retest reliability was good to excellent across the four subscales and was generally stronger than that reported for the Korean MPOC-SP, particularly for PGI [15]. Nevertheless, the small systematic decreases observed for SIS and CSI indicate that high ICC values should not be equated with complete temporal stability, because ICC primarily reflects the relative consistency of individuals’ scores and does not exclude systematic change between measurements [26,27,30]. In addition, small baseline differences in TPR and CSI between participants who did and did not complete the retest suggest that complete representativeness of the retest subsample cannot be assumed. These considerations warrant some caution when interpreting the test–retest findings.

The SEM and MDC95 findings provide complementary information regarding measurement error beyond relative reliability [26,30,31]. Although these estimates can help distinguish observed change from expected measurement variability, their magnitude cannot be classified as clinically acceptable or meaningful in the absence of an established external criterion or minimal important change value for the MPOC-SP. This distinction is particularly important when considering the use of the instrument for repeated assessment. SEM and MDC95 characterize measurement error and should not be interpreted as evidence of responsiveness or as thresholds for meaningful change [24,30,31]. Longitudinal studies incorporating appropriate external anchors are therefore needed to establish responsiveness and meaningful change thresholds for the Turkish MPOC-SP.

The factor-analytic findings supported the hypothesized four-factor structure of the Turkish MPOC-SP, with strong item–factor relationships and satisfactory overall model fit. This is consistent with the Spanish adaptation, which also reported support for the specified MPOC-SP factor structure [13]. The moderate correlations among the four factors, together with the CR, AVE, Fornell–Larcker, and HTMT findings, were consistent with the interpretation of SIS, TPR, CSI, and PGI as related but distinguishable dimensions [32,33,34]. These model-based findings provide complementary evidence regarding the internal measurement structure and factor distinctiveness of the Turkish MPOC-SP. They should not, however, be interpreted as evidence of external convergent or discriminant validity, because no independent measure of a comparable service provider–reported construct was included.

No substantial floor or ceiling effects were observed at the subscale level. However, 14 of the 27 items exceeded the predefined 15% criterion for a ceiling effect, indicating some clustering of responses at the upper end of the scale at the item level. Although this pattern did not substantially affect the distributions of the subscale scores, it may reduce the ability of some items to discriminate among service providers reporting very high levels of FCS delivery and to capture further improvement at the upper end of the scale [35]. Because the MPOC-SP is a self-report measure of professionally desirable service behaviors, self-report or social desirability tendencies may have contributed to the generally high scores and item-level ceiling effects. However, no independent observational or external measure was included to evaluate this possibility, and the observed ceiling effects therefore cannot be attributed specifically to social desirability.

The exploratory institutional analyses identified differences only for PGI, with higher scores observed in private practice than in some other institutional settings. Although the magnitude of these differences varied across pairwise comparisons, the pattern is of interest because PGI addresses the provision of general and written information, community resources, and support to families. Previous MPOC-SP studies have similarly identified general information as an area in which FCS delivery may be less consistently achieved [10,36]. Interpretation of the present institutional differences nevertheless requires caution because professional disciplines were unevenly distributed across settings. Although the PGI difference persisted after adjustment for profession, age, and professional experience, residual confounding cannot be excluded. More importantly, no independent criterion or a priori evidence established that the institutional groups were expected to differ in family-centered service delivery. Accordingly, these findings should be regarded as exploratory institutional differences rather than evidence of known-groups validity.

The present study extends the FCS measurement framework currently available in Türkiye. Previous research established the Turkish versions of the MPOC-20 and MPOC-56, which evaluate families’ perceptions of FCS delivery [22]. The Turkish MPOC-SP complements these instruments by providing a standardized assessment of service providers’ perceptions of these processes. Previous research has emphasized the value of considering both parent and service-provider perspectives, as differences may exist between the services professionals perceive themselves as providing and those experienced by families [10]. The availability of the Turkish MPOC-20, MPOC-56, and MPOC-SP therefore provides an opportunity to evaluate FCS delivery from complementary family and provider perspectives. However, agreement between the Turkish MPOC-SP and parent-reported MPOC instruments was not examined in the present study. Importantly, interpretation of the MPOC-SP should focus on its four established subscales rather than on an overall total score.

In addition to its measurement properties, the cultural adaptation process supports the linguistic and conceptual appropriateness of the Turkish MPOC-SP. The translation and adaptation procedures followed established cross-cultural adaptation principles, with only minor linguistic modifications required to improve clarity and cultural appropriateness while maintaining conceptual equivalence [23]. Cross-cultural adaptation requires more than literal translation and should preserve the intended meaning of items within the cultural and service context of the target population [23]. The present findings indicate that the adapted items functioned coherently within the proposed four-factor structure. Nevertheless, formal measurement invariance analysis would be required before equivalence across professional, institutional, or cultural groups could be established.

The MPOC-SP has been applied across different pediatric service contexts, supporting the relevance of a culturally adapted Turkish version [36,37,38]. From a service-evaluation perspective, the Turkish MPOC-SP may provide a structured means of examining distinct dimensions of FCS and identifying potential areas for improvement [10,36]. These processes are also conceptually consistent with the ICF emphasis on functioning and participation within the child’s broader environmental and family context [5,6,7,39]. However, because responsiveness and meaningful change thresholds have not yet been established, longitudinal changes in Turkish MPOC-SP scores should not be interpreted as clinically or practically meaningful improvement.

Several limitations should be considered when interpreting the findings of this study. First, although participants represented multiple professional disciplines, institutional settings, and 15 provinces of Türkiye, recruitment was based on nonprobability sampling rather than a nationally representative population-based sampling frame. Moreover, 149 participants (55.8% of the sample) were recruited from Istanbul, Ankara, and Izmir, indicating substantial geographical concentration despite recruitment across 15 provinces. Approximately 450 potentially eligible service providers were approached, and 283 responded; however, the resulting proportion of 62.9% should be interpreted as the response proportion among those approached rather than as a population-level response rate. Because information on nonrespondents was not available, potential selection and nonresponse bias cannot be excluded. In addition, professional groups and institutional settings were not equally represented, and more than half of the participants worked in special education and rehabilitation centers. Therefore, the findings should not be interpreted as nationally representative estimates of family-centered service practices among pediatric service providers in Türkiye, and generalization to individual professional groups should be made cautiously.

Second, the MPOC-SP is a self-report measure reflecting service providers’ perceptions of their own family-centered practices. Responses may therefore have been influenced by social desirability, professional self-perception, and recall bias. Moreover, self-reported practices may not correspond to actual family-centered behaviors observed in clinical or service-delivery settings. This limitation is particularly relevant because no family-reported MPOC measure, observational assessment, or external service-quality criterion was administered in the present study. Although no substantial ceiling effects were observed at the subscale level, the observed item-level ceiling effects may reduce discrimination among respondents reporting very high levels of FCS delivery.

Third, although the test–retest subsample was generally comparable to participants who completed only the baseline assessment, small baseline differences were observed for TPR and CSI. Therefore, complete representativeness of the test–retest subsample cannot be assumed. In addition, the small systematic decreases observed for SIS and CSI should be considered when interpreting repeated measurements.

Fourth, external convergent validity could not be directly examined because, to our knowledge, no validated Turkish-language instrument measuring a sufficiently comparable service provider–reported construct of family-centered service delivery was available as an appropriate external comparator. Criterion validity was also not evaluated because no established gold-standard measure exists for the construct assessed by the MPOC-SP. Consequently, the model-derived CR, AVE, Fornell–Larcker, and HTMT findings should be interpreted as supplementary information regarding the internal measurement model rather than as substitutes for external construct validation.

Fifth, the institutional-group comparisons were exploratory. No independent criterion was available to establish a priori differences in FCS delivery across institutional settings, and professional disciplines were not evenly distributed across settings. Although additional analyses adjusted for profession, age, and years of professional experience, residual confounding cannot be excluded. Therefore, the observed institutional differences should not be interpreted as evidence of known-groups validity.

Sixth, measurement invariance across professional groups, institutional settings, or other participant subgroups was not evaluated because several subgroups were relatively small and group sizes were unequal. Consequently, it remains uncertain whether the four-factor structure and item parameters operate equivalently across these subgroups.

Finally, responsiveness and clinically meaningful change were not evaluated. SEM and MDC95 were calculated to characterize measurement error and should not be interpreted as evidence of responsiveness or as thresholds for clinically important change [24,30,31]. Future studies should examine external construct validity, responsiveness, clinically meaningful change, and measurement invariance in larger and more balanced samples and should further evaluate the Turkish MPOC-SP in independently recruited service-provider populations.

In conclusion, the Turkish version of the MPOC-SP demonstrated high internal consistency and good to excellent test–retest reliability, while the factor-analytic findings were consistent with the hypothesized four-factor structure of the original instrument. The findings provide initial evidence supporting the interpretation of the Turkish MPOC-SP at the level of its four established subscales rather than as an overall total score. Together with the Turkish versions of the MPOC-20 and MPOC-56, the Turkish MPOC-SP provides an opportunity to evaluate FCS delivery from complementary family and service-provider perspectives. The instrument has potential for evaluating FCS processes across pediatric healthcare, rehabilitation, and educational settings in Türkiye; however, its applicability across these settings requires further independent validation. Further studies in independent and more balanced samples are needed to establish external construct validity, measurement invariance, responsiveness, and clinically meaningful change.

## Figures and Tables

**Table 1:** Demographic, professional, and geographical characteristics of the participants.

Characteristic	n (%) or Mean ± SD
**Age (years)**	33.13 ± 8.00
**Professional experience (years)**	10.17 ± 7.84
**Sex**	
Female	199 (74.5)
Male	68 (25.5)
**Profession**	
Physiotherapist	57 (21.3)
Occupational therapist	51 (19.1)
Speech and language therapist	56 (21.0)
Psychologist	27 (10.1)
Special education teacher/child development specialist	26 (9.7)
Physician	23 (8.6)
Nurse	27 (10.1)
**Institution**	
Special education and rehabilitation center	146 (54.7)
Public hospital	60 (22.5)
Private hospital	34 (12.7)
Private practice	27 (10.1)
**Province**	
Istanbul	61 (22.8)
Ankara	59 (22.1)
Izmir	29 (10.9)
Bursa	16 (6.0)
Antalya	14 (5.2)
Konya	12 (4.5)
Adana	12 (4.5)
Kocaeli	10 (3.7)
Şanlıurfa	9 (3.4)
Gaziantep	9 (3.4)
Diyarbakır	8 (3.0)
Mersin	8 (3.0)
Hatay	7 (2.6)
Trabzon	7 (2.6)
Giresun	6 (2.2)

n, number; SD, standard deviation.

**Table 2:** Item-level descriptive statistics and internal consistency of the Turkish version of the Measure of Processes of Care for Service Providers (MPOC-SP).

Subscale	Item	n	Mean ± SD	Floor Effect (%)	Ceiling Effect (%)	Corrected Item–Subscale Correlation	Cronbach’s α If Item Deleted	McDonald’s ω
SIS	1	267	5.52 ± 1.22	0.75	20.22	0.863	0.964	—
	2	267	5.61 ± 1.22	0.00	25.09	0.876	0.964	—
	3	267	5.50 ± 1.25	0.75	19.48	0.877	0.964	—
	4	267	5.19 ± 1.23	1.12	8.99	0.822	0.966	—
	5	267	5.59 ± 1.24	0.75	23.60	0.872	0.964	—
	8	267	5.46 ± 1.12	0.00	14.98	0.807	0.966	—
	9	267	5.43 ± 1.14	0.00	14.23	0.829	0.965	—
	11	267	5.50 ± 1.15	0.00	18.35	0.860	0.964	—
	12	267	5.48 ± 1.16	0.37	14.61	0.895	0.963	—
	21	267	5.54 ± 1.11	0.00	19.48	0.821	0.966	—
SIS subscale score	—	267	5.48 ± 1.04	0.00	2.25	—	0.968	0.968
TPR	6	267	5.76 ± 0.93	0.00	21.35	0.749	0.936	—
	7	267	5.33 ± 0.87	0.00	6.37	0.710	0.938	—
	10	267	5.75 ± 0.85	0.00	17.98	0.766	0.935	—
	13	267	5.76 ± 0.91	0.00	20.97	0.790	0.933	—
	17	267	5.76 ± 0.89	0.00	19.48	0.832	0.931	—
	18	267	5.57 ± 0.85	0.00	10.11	0.761	0.935	—
	19	267	5.63 ± 0.85	0.00	12.73	0.776	0.934	—
	20	267	5.70 ± 0.88	0.00	16.85	0.807	0.932	—
	22	267	5.76 ± 0.93	0.00	22.85	0.770	0.934	—
TPR subscale score	—	267	5.67 ± 0.73	0.00	0.37	—	0.941	0.941
CSI	14	267	5.53 ± 1.14	0.00	17.60	0.813	0.836	—
	15	267	5.21 ± 1.19	0.37	9.36	0.786	0.860	—
	16	267	5.69 ± 1.14	0.37	23.60	0.787	0.859	—
CSI subscale score	—	267	5.48 ± 1.05	0.00	5.62	—	0.896	0.896
PGI	23	267	5.07 ± 1.36	2.25	8.61	0.684	0.868	—
	24	267	5.30 ± 1.27	1.12	12.73	0.746	0.854	—
	25	267	5.03 ± 1.46	3.37	8.99	0.779	0.845	—
	26	267	5.27 ± 1.34	1.87	14.61	0.697	0.865	—
	27	267	5.32 ± 1.27	1.87	13.48	0.706	0.863	—
PGI subscale score	—	267	5.20 ± 1.11	0.37	1.12	—	0.884	0.885

For individual item rows, Cronbach’s α values represent α if the respective item was deleted; for subscale score rows, Cronbach’s α and McDonald’s ω represent the internal consistency coefficients for the complete subscale. CSI, Communicating Specific Information; n, number; PGI, Providing General Information; SIS, Showing Interpersonal Sensitivity; SD, standard deviation; TPR, Treating People Respectfully; —: Not applicable.

**Table 3:** Test–retest reliability and measurement-error estimates of the Turkish MPOC-SP.

Subscale	Test Mean ± SD	Retest Mean ± SD	ICC(2,1) (95% CI)	Mean Difference (Test − Retest) (95% CI)	*p*	SEM	MDC95
SIS	5.39 ± 1.27	5.26 ± 1.09	0.963 (0.932–0.979)	0.129 (0.065–0.193)	<0.001	0.228	0.633
TPR	5.79 ± 0.85	5.76 ± 0.67	0.819 (0.718–0.885)	0.026 (−0.073–0.125)	0.601	0.326	0.903
CSI	5.66 ± 1.19	5.44 ± 1.09	0.918 (0.858–0.951)	0.213 (0.124–0.303)	<0.001	0.328	0.908
PGI	5.32 ± 1.12	5.27 ± 0.97	0.952 (0.922–0.971)	0.042 (−0.027–0.111)	0.231	0.228	0.633

CSI, Communicating Specific Information; ICC, intraclass correlation coefficient; MDC95, minimal detectable change at 95% confidence level; PGI, Providing General Information; SIS, Showing Interpersonal Sensitivity; SEM, standard error of measurement; SD, standard deviation; TPR, Treating People Respectfully.

**Table 4:** Confirmatory factor analysis fit indices for the original four-factor model.

Fit Index	Value
χ^2^	463.24
df	318
χ^2^/df	1.46
CFI	0.998
TLI	0.998
RMSEA	0.041
RMSEA 90% CI	(0.033–0.049)
SRMR	0.057

CFI, comparative fit index; CI, confidence interval; df, degrees of freedom; RMSEA, root mean square error of approximation; SRMR, standardized root mean square residual; TLI, Tucker–Lewis index; χ^2^, chi-square; χ^2^/df, chi-square-to-degrees-of-freedom ratio.

**Table 5:** Standardized factor loadings, standard errors, and 95% confidence intervals for the four-factor Turkish MPOC-SP.

Subscale	Item	Standardized Loading	Standard Error	95% Confidence Interval
SIS	1	0.901	0.009	0.884–0.918
	2	0.933	0.008	0.917–0.949
	3	0.905	0.008	0.888–0.922
	4	0.837	0.010	0.817–0.857
	5	0.918	0.008	0.902–0.934
	8	0.854	0.010	0.834–0.873
	9	0.875	0.010	0.856–0.894
	11	0.910	0.009	0.894–0.927
	12	0.929	0.008	0.913–0.945
	21	0.877	0.010	0.858–0.896
TPR	6	0.807	0.013	0.781–0.834
	7	0.761	0.014	0.732–0.789
	10	0.870	0.013	0.844–0.895
	13	0.863	0.013	0.838–0.887
	17	0.901	0.012	0.878–0.0925
	18	0.826	0.013	0.801–0.852
	19	0.878	0.013	0.853–0.903
	20	0.909	0.012	0.885–0.933
	22	0.842	0.013	0.816–0.867
CSI	14	0.926	0.017	0.892–0.960
	15	0.804	0.017	0.771–0.838
	16	0.889	0.017	0.855–0.922
PGI	23	0.812	0.019	0.775–0.849
	24	0.824	0.018	0.788–0.860
	25	0.811	0.018	0.776–0.847
	26	0.786	0.019	0.748–0.823
	27	0.766	0.019	0.728–0.804

CSI, Communicating Specific Information; PGI, Providing General Information; SIS, Showing Interpersonal Sensitivity; TPR, Treating People Respectfully.

**Table 6:** (**A**) Composite reliability and model-based indices of factor distinctiveness. (**B**) Latent factor correlations, 95% bootstrap confidence intervals, and square roots of average variance extracted (AVE).

(**A**)						
**Subscale**	**CR**		**AVE**		**√AVE**	
SIS	0.976		0.800		0.894	
TPR	0.960		0.726		0.852	
CSI	0.907		0.765		0.875	
PGI	0.899		0.640		0.800	
(**B**)						
**Subscale**	**SIS**	**TPR**		**CSI**		**PGI**
SIS	0.894					
TPR	0.385 (0.277–0.483)	0.852				
CSI	0.379 (0.271–0.478)	0.679 (0.611–0.739)		0.875		
PGI	0.420 (0.316–0.517)	0.525 (0.434–0.608)		0.642 (0.567–0.709)		0.800

(**A**) AVE, average variance extracted; CR, composite reliability; CSI, Communicating Specific Information; PGI, Providing General Information; SIS, Showing Interpersonal Sensitivity; TPR, Treating People Respectfully. (**B**) Diagonal values represent the square roots of AVE; off-diagonal values represent latent factor correlations with approximate 95% bootstrap confidence intervals in parentheses. Confidence intervals were obtained using a parametric model-based bootstrap with 20,000 replications. CSI, Communicating Specific Information; PGI, Providing General Information; SIS, Showing Interpersonal Sensitivity; TPR, Treating People Respectfully.

**Table 7:** Exploratory comparison of Turkish MPOC-SP subscale scores across institutional settings.

Subscale	Special Education and Rehabilitation Center (n = 146)	Public Hospital (n = 60)	Private Hospital (n = 34)	Private Practice (n = 27)	H	*p*	ε^2^
SIS	5.70 (5.13–6.20)	5.40 (4.48–6.10)	5.90 (5.10–6.30)	5.70 (5.30–6.55)	5.875	0.118	0.011
TPR	5.78 (5.33–6.22)	5.67 (4.89–6.22)	5.72 (5.17–6.00)	5.67 (5.33–6.28)	1.876	0.599	0.000
CSI	5.67 (5.00–6.00)	5.67 (5.00–6.33)	5.67 (4.75–6.00)	5.67 (5.33–6.17)	1.847	0.605	0.000
PGI	5.40 (4.60–6.00)	5.20 (4.00–5.80)	5.50 (4.80–6.00)	6.00 (5.50–6.40)	16.841	<0.001	0.053

Values are presented as median (interquartile range). CSI, Communicating Specific Information; ε^2^, epsilon-squared; H, Kruskal–Wallis test statistic; n, number; PGI, Providing General Information; SIS, Showing Interpersonal Sensitivity; TPR, Treating People Respectfully.

**Table 8:** Cross-tabulation of professional discipline by institutional setting.

Profession	Special Education and Rehabilitation	Public Hospital	Private Hospital	Private Practice	Total
Physiotherapist	24	16	10	7	57
Occupational therapist	42	5	1	3	51
Speech and language therapist	34	7	6	9	56
Psychologist	23	1	1	2	27
Special education/child development specialist	23	1	1	1	26
Physician	0	13	5	5	23
Nurse	0	17	10	0	27
Total	146	60	34	27	267

## Data Availability

The data presented in this study are available on request from the corresponding author due to ethical reasons.

## Abbreviations

The following abbreviations are used in this manuscript:

AVE	Average Variance Extracted
CFA	Confirmatory Factor Analysis
CFI	Comparative Fit Index
CI	Confidence Interval
CR	Composite Reliability
CSI	Communicating Specific Information
FCS	Family-Centered Services
ICC	Intraclass Correlation Coefficient
MDC95	Minimal Detectable Change at the 95% Confidence Level
MPOC	Measure of Processes of Care
MPOC-SP	Measure of Processes of Care for Service Providers
PGI	Providing General Information
RMSEA	Root Mean Square Error of Approximation
SEM	Standard Error of Measurement
SIS	Showing Interpersonal Sensitivity
SRMR	Standardized Root Mean Square Residual
TLI	Tucker–Lewis Index
TPR	Treating People Respectfully
